# Simultaneous microRNA-612 restoration and 5-FU treatment inhibit the growth and migration of human PANC-1 pancreatic cancer cells

**DOI:** 10.17179/excli2020-2900

**Published:** 2021-01-21

**Authors:** Darya Javadrashid, Reza Mohammadzadeh, Amir Baghbanzadeh, Sahar Safaee, Mohammad Amini, Ziba Lotfi, Elham Baghbani, Vahid Khaze Shahgoli, Behzad Baradaran

**Affiliations:** 1 Immunology Research Center, Tabriz University of Medical Sciences, Iran; 2 Faculty of Basic Sciences, Department of Biology, University of Maragheh, Iran

**Keywords:** miR-612, PANC-1, 5-FU, pancreatic cancer, electroporation

## Abstract

Despite the recent advances in the treatment of other cancers, the 5-year survival rate of pancreatic cancer remains under 9 %. Chemotherapy and surgical resection are the most common therapy methods. The regulatory role of microRNAs in different types of cancer has given them therapeutic importance. miR-612 has been downregulated in colorectal, bladder, liver, and some other types of cancer and could be considered a tumor-suppressor miRNA. 5-FU is one of the most common chemotherapeutic agents used in pancreatic cancer treatment, which is used in multiple drug regimens and combinatorial therapy methods. The aim of this study is the evaluation of miR-612 restoration in the PANC-1 cell line and using the tumor-suppressive effect of it in combination with 5-FU on cell growth and migration. MiR-612 mimic was transfected to PANC-1 cells through electroporation. Following the transfection, expression levels of miR-612 and BAX, BCL-2, Caspase-3, MMP9, and PD-L1 genes were measured by qRT-PCR. MTT assay was used to determine the cytotoxicity of miR-612 and 5-FU on PANC-1 cell viability. To confirm MTT results and to evaluate the quantitative effect of apoptosis induction flow cytometry test was used and in order to confirm apoptosis test results and cell cycle arrest evaluation DAPI staining and cell, cycle tests were conducted, respectively. Finally, to assess the inhibitory effect of miR-612 in combination with 5-FU on migration and growth wound healing and colony formation assays were used, respectively. Results demonstrated that miR-612 alongside 5-FU has an important role in the inhibition of migration and growth and also apoptosis induction in PANC-1 cells and could be considered as a supporting agent of chemotherapy and a novel therapeutic modality in pancreatic cancer treatment.

## Introduction

Pancreatic ductal adenocarcinoma is certainly one of the most challenging and lethal human malignancies (Steele et al., 2019[23]) . It is expected that pancreatic cancer is going to be the second-leading cause of cancer-related death by the end of the next decade (Rahib et al., 2014[17]). The 10-year overall survival rate still stays poor for the patients after surgical resection (4 %) (Paniccia et al., 2015[16]). The most frequent type of pancreatic cancer is pancreatic ductal adenocarcinoma which covers over 90 % of pancreatic tumors (Hidalgo et al., 2015[9]). The speed of its progression, metastasis, and the lack of favorable prognostic biomarkers are several factors that decrease the 5-year survival rate of this cancer to less than 10 %. Also, chemo-therapeutic resistance caused by the pancreatic tumor microenvironment, disease recurrence after surgical resection, and metastasis to other organs such as the liver are further factors that turn the treatment of this cancer to more of a challenge (Dauer et al., 2017[6]).

Despite advances in the treatment modalities, systemic chemotherapy has the most efficiency on survival rate, and from the time of Gemcitabine (GEM) approval as the first treatment strategy in 1997, several new multiple regimen system is being evaluated in the treatment of pancreatic cancer (Burris et al., 1997[4]). The four regimen system consisting of 5-fluorouracil, epirubicin, cisplatin, and gemcitabine has shown a better overall survival compared to GEM alone (Reni et al., 2005[18]). In 2011, a four regimen system named FOLFIRINOX (folinic acid, 5-FU, irinotecan, and oxaliplatin) showed a significantly better survival rate compared to GEM alone (Conroy et al., 2011[5]). Yet, the outcomes of therapy for pancreatic cancer remain stagnant and researchers seek a favorable method to improve the patients' health. Combination therapy is one of the strategies that might be helpful to this matter (Hajatdoost et al., 2018[8]).

MicroRNAs have attracted researchers' attention due to their regulatory roles in different physiological processes from which their part in cancer initiation and progression is the most significant (Davis-Dusenbery and Hata, 2010[7]; Baghbanzadeh et al., 2020[2]; Kooshkaki et al., 2020[12]; Shabani et al., 2019[19]; Baradaran et al., 2019[3]). These small non-coding RNAs have been used as therapeutic methods and diagnostic biomarkers. Numerous studies have investigated approaching technologies to inhibit these molecules. During recent years, tumor suppressor miRNAs, and microRNA replacement therapy in cancer cells have come into notice (Bader et al., 2010[1]). miR-612 has shown tumor-suppressive activity in various cancers such as hepatocellular carcinoma, melanoma, bladder, colorectal, ovarian, cervical, and non-small cell lung cancer (Liu et al., 2016[15]; Sheng et al., 2015[20]; Liu et al., 2018[14]; Zhu et al., 2018[28]; Yu et al., 2018[27]; Kang et al., 2019[11]; Jin et al., 2020[10]). 

Since the combination therapy seems a more promising modality in the treatment of cancer, here in this study we decided to evaluate the simultaneous effect of miR-612 and chemo-therapeutic agent 5-FU on the inhibition of growth and migration of PANC-1 human pancreatic cancer cell line.

## Materials and Methods

### Cell culture

Cell lines of human pancreatic cancer PANC-1 and Mia-PaCa2 were purchased from the Pasture Institute's Cell Bank (Tehran, Iran). The RPMI-1640 culture media (Gibco, USA, NYC) was used to maintain the cells alongside with 10 % fetal bovine serum (FBS) (Gibco, USA, NYC) supplementation, 1 % Pen-Strep antibiotics (100 IU/ml penicillin, 100 mg/ml streptomycin) (Gibco, USA, NYC). The cells were stored in an incubator at 37 °C, 95 % of humidity, and 5 % of carbon dioxide (CO_2_) atmosphere. Culture medium refreshment was applied nearly every day and the cells were passaged using 0.25 % trypsin/EDTA solution treatment when reaching an 80-90 % confluence.

### RNA extraction and qRT-PCR

Primer sequences were readily ordered from Bioneer (South Korea), as demonstrated in Table 1(Tab. 1). Two different pancreatic cancer cell lines (PANC-1 and Mia PaCa-2) were cultured and the cells were seeded in 6-well plates (SPL, South Korea) at the density of 6×10^5^ cells per well. Afterward, Trizol reagent (RiboEx Kit, GeneAll, South Korea) was used for total RNA extraction and cDNA synthesis was conducted by its specific kit (Biofact, South Korea) according to the instructions. To define the expression level of miR-612, the specified cDNA synthesis kit (BonMir, Iran) was used.

Quantitative real-time PCR by SYBR Premix Ex Taq (Biofact) via StepOne Plus real-time PCR system (Applied Biosystems, Thermo Fisher Scientific, USA) was exploited to assess the expression levels of BAX, BCL-2, Caspae-3, MMP-9, and PDL-1 genes. GAPDH and SNORD were considered as control genes for target genes and detection of miR-612, respectively.

### Electroporation of miRNA

Prior to electroporation, and to continue transfection experiments, PANC-1 as the lowest expressing miR-612 cell line was selected. PANC-1 cells were seeded in a complete media in six-well plates and at the 6×10^5^ cells per well density per well and were electroporated with three different concentrations of 80, 100, and 120 pmol of miR-612 using Gene Pulser Xcell (Bio-Rad, USA). After three different time spans of incubation (24, 48, and 72 hours), qRT-PCR was used to measure the relative expression of miR-612. Among the aforementioned concentrations, the one with the maximum fluctuations was chosen for the rest of the project experiments.

### Proliferation assay

miR-612 and 5-FU related cytotoxicity was measured by 3‐(4,5‐dimethylthiazol‐2‐yl)‐2,5-diphenyltetrazolium bromide (MTT) (Sigma‐Aldrich, Germany). For 24 hours, cells were seeded at the density of 8×10^3 ^cells per well in 96-well culture plates (SPL, Korea). Thereafter, the seeded cells were divided into four experimental groups consisting of the negative control (untreated and untransfected), transfected with miR-612, treated by 5-FU, and treated by 5-FU and transfected with miR-612 simultaneously. The transfection and treatment were conducted by the following order. After 24 hours of incubation following the miR-612 transfection, the cells were treated with two different concentrations of 5-FU chemotherapeutic agents of IC_25_ and IC_50_. Consequently, cells were incubated for 24 hours at 37 °C, 95 % of humidity, and 5 % CO_2_ overnight. Following the overnight incubation, 100 μL culture medium with 50 μL of MTT (2 mg/mL in phosphate‐buffered saline [PBS]) was added to each well after removal of the culture medium and then incubated for an extra 4 hours at 37 °C.

200 μL of dimethyl sulfoxide (DMSO) (Sigma‐Aldrich) was added to each well to dissolve the formazan crystals. The ocular density of each well was measured at a wavelength of 570 nm using an ELISA reader (Tecan, Switzerland) after 30 minutes of incubation. A triplicate method was used to conduct all experiments.

### Annexin V/PI stain (Apoptosis assay)

In order to achieve apoptosis induction rate quantitatively, an annexin‐V‐FITC and propidium iodide (PI) double staining kit (Exbio, Czech Republic) was used. Cells were seeded at a density of 6×10^5^ cells per well in six-well plates. At that point, wells were grouped as untransfected and untreated), transfected with miR-612, treated by 5-FU, and transfected and treated by miR-612 and 5-FU simultaneously. After 48 hours of incubation (the optimal time for miRNA), the cells were detached by trypsin, washed by PBS thrice, suspended in 500 μL of binding buffer, and then incubated with 5 μL of FITC‐conjugated annexin‐V and 5 μL of PI for 15 minutes at room temperature in complete darkness. The stained cells were detected by flow cytometry (FACSQuant; Milteny, Germany) and FlowJo software version 7.6 (FlowJo; flowjo.com) was used to analyze the gathered data.

### DAPI stating

To achieve apoptosis induction quality 4′,6-diamidino-2-phenylindole (DAPI), which is a fluorescent color that attaches to AT-rich regions in the DNA strands, was used. Different morphologies of the cells such as apoptotic bodies and nucleus, chromosome accumulation, and segmentation are detectable using this method. The cells were seeded at 96-well plates at the density of 8×10^3^ cells per well and grouped into control, transfected with miR-612, treated by 5-FU, and transfected and treated by miR-612 and 5-FU simultaneously. Following 24 hours of miR-612 transfection, 5-FU treatment was conducted and after further 24 hours of incubation, the stating protocol was performed. Wells were washed by PBS and 100 μl of Paraformaldehyde was added to each of them following an hour of incubation. Then, after another wash by PBS, 100 μl of 0.01 % Triton was added to each well and the plate was incubated for another 15 minutes. Afterward, followed by another PBS wash, 100 μl of DAPI solution was added to each well in the dark. 10 further minutes of dark incubation and 200 μl of PBS addition to each well, the results were analyzed and captured using Cytation 5 imaging reader (Biotech, USA).

### Cell cycle analysis

The cells were seeded at the same density for apoptosis assay and RNase A was used to eliminate RNA molecules. The cells were grouped in the same fashion used for other tests. Using Trypsin/EDTA solution, the cells were detached from the 6-well plates and were washed by cold PBS twice. Then, 1 ml of 75 % ethanol was used for each group of the cells in order to fixation and incubated at -20 °C overnight. The following day, cells were washed by PBS one more time and 5 μl of RNase A was added to each group and incubated for 30 minutes at 37 °C. Afterward, 20 μl PI alongside Triton X100 was added to the cells. Cell cycle arrest was detected with the flow cytometry and analyzed by FlowJo software as well.

### Wound-healing assay (scratch)

Wound healing assay (scratch test) was used for measuring the migration of the cells. Cells (2×10^5^ cells per well) were seeded for 24 hours in 24‐well plates (the transfected groups already transfected with miR-612). After 24 hours of incubation, in order to mimic a wound, a scratch was made in the center of each well using a yellow pipette tip across the cell monolayer to create an open gap. The wells were washed with PBS to remove the cell debris. As the next step, the drug control considered group was treated by 5-FU and the other wells were considered as control groups. Using an inverted microscope (Optica, Italy), pictures from each well were taken at the time spans of 0, 24, 48, and 72 hours (cells were incubated between the time spans at 37 °C and 5 % CO_2_ overnight in order to allow migration). An inverted microscope was used to capture images of the gaps (wounds) at 0, 24, 48, and 72 hours. The migration rate was enumerated by computing the gap between the wound edges by using ImageJ software. Three independent repetitions were performed for this assay.

### Colony formation

To evaluate the effect of 5-FU and miR-612 on the colonies initiated by the cancer cells *in vivo*, the colony formation assay was used. The cells were cultured at the density of 10^3 ^cells per well in a 6-well plate and grouped by the following order: control group, miR-612 transfected group, 5-FU treated group, and the combination group. After incubation for 3 to 4 days, the plate was investigated for colonies formed during that time. Reaching the ideal colony formation situation, the wells were washed by PBS and stained by 1 ml crystal violet (Sigma Aldrich, USA) and kept in the room temperature for 30 minutes. Washed by PBS one more time, the images were taken by both regular and microscopic cameras.

### Statistical analysis

At least three repetitions from three independent tests have been performed for each and every one of the data gathered in this experiment. Continuous variables were expressed as the mean ± standard deviation (SD). Analysis of variance, one-way analysis of variance (ANOVA) and two-way ANOVA followed by Dunnett's test, was used to determine the significant differences between the groups. All statistical analyses were completed using GraphPad Prism version 6.01. P<0.05 was considered statistically significant.

## Results

### miR-612 was downregulated in PANC-1 cell line

**Figure 1:**
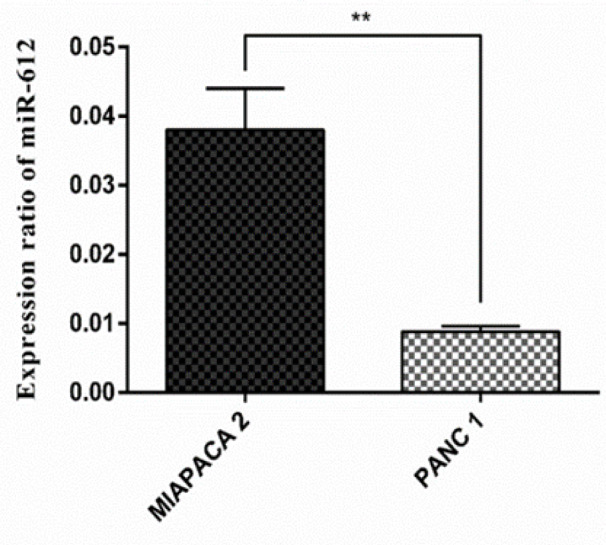
The relative expression level of miR-612 in pancreatic cancer cell lines in comparison (**P<0.01).

As it is demonstrated in Figure 1(Fig. 1), after measuring miR-612 expression in two different pancreatic cancer cell lines, due to the significant downregulation of miR-612 in the PANC-1 cell line compared to Mia PaCa-2 cells, PANC-1 was selected as the elected cell line for the rest of the experiments (P < 0.01).

### Ideal concentration and effective time span for miR-612 was perceived after qRT-PCR

**Figure 2:**
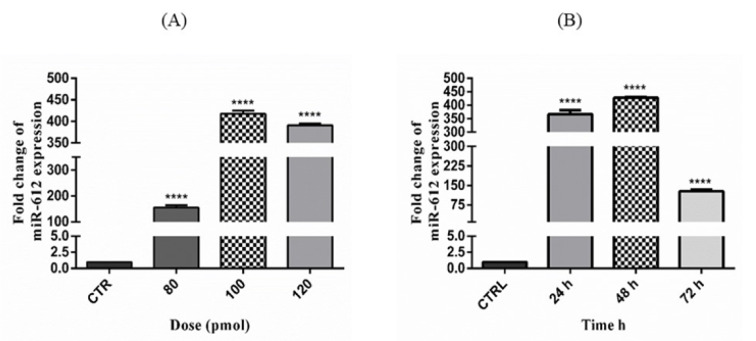
(A) The selected concentration of miR-612 mimic (****P<0.0001). (B) Ideal time span of miR-612 mimic transfection to PANC-1 cell line (****P<0.0001).

**Figure 3:**
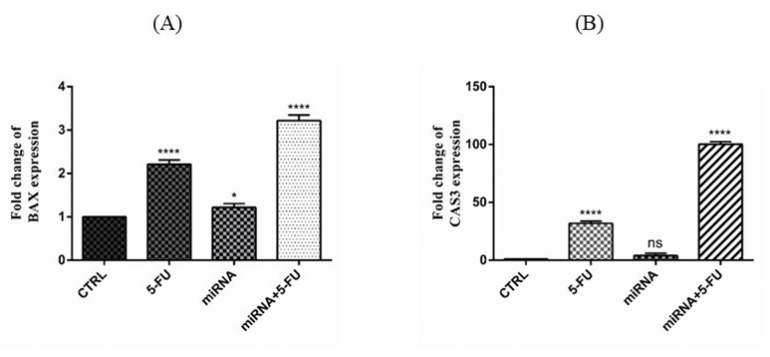
(A) Relative expression of BAX gene after miR-612 mimic regulation and 5-FU treatment in PANC-1 cells. Both miR-612 and combination groups show a significant increase in fold change of BAX expression (****P<0.0001). (B) Relative expression of Caspase 3 gene after miR-612 mimic regulation and 5-FU treatment. As seen in the picture, expression change after miR-612 transfection is non-significant and the increase after 5-FU treatment both solo and in combination with 5-FU is significant (****P<0.0001).

After miR-612 transfection evaluation using qRT-PCR in three different concentrations and time spans, the results were as shown in Figures 2(Fig. 2) and 3(Fig. 3). The concentration of 100 pmol of miR-612 mimic induced the highest fold changes of miR-612 expression and the 48 hours had the highest transfection efficacy within.

### Upregulation of miR-612 using mimic transfection altered the expression of some apoptotic, anti-apoptotic, metastatic and immune checkpoint genes (BAX, Cas-3, BCL-2, MMP9, and PD-L1)

The effect of miR-612 mimic regulation alongside 5-FU on the expression of target genes was determined by SYBR Green qRT-PCR. As shown in Figure 3(Fig. 3), both apoptotic genes BAX and Caspase 3 were significantly upregulated in the miR-612 and 5-FU combination groups.

**Figure 4:**
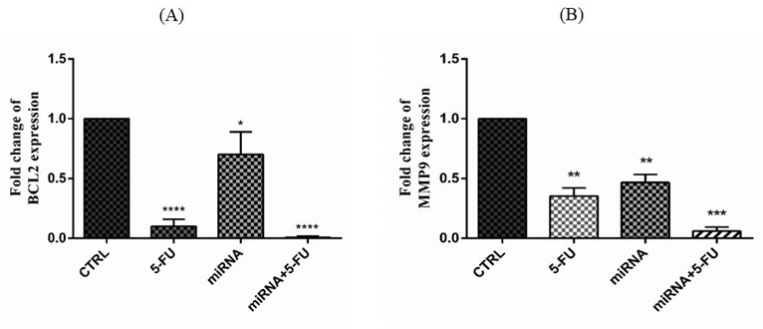
(A) Relative expression of BCL-2 gene after miR-612 mimic regulation and 5-FU treatment in PANC-1 cells. Both 5-FU and combination groups show a significant decrease in fold change of BCL-2 expression (****P<0.0001) and miR612 group shows rather significant decrease as well (*P<0.05). (B) Relative expression of MMp9 gene after miR-612 mimic regulation and 5-FU treatment. As seen in the picture, expression change after both miR-612 transfection and 5-FU treatment alone is significant (**P<0.01) and the decrease in the combination group is even more significant (***P<0.001).

The expression changes in BCL-2 (anti-apoptotic) and MMP9 (metastatic) genes were rather different than the previous genes. Both of them were downregulated after transfection of miR-612 and 5-FU treatment as demonstrated in Figure 4(Fig. 4).

**Figure 5:**
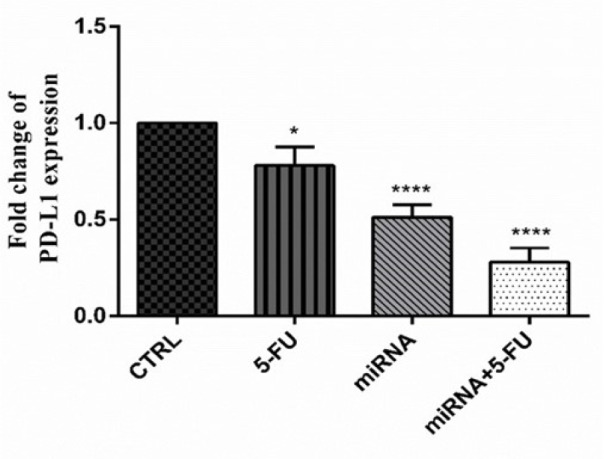
Fold change of PD-L1 expression after transfection of miR-612 mimic and 5-FU treatment. As it can be seen in the picture the change is significant in 5-FU treated (*P<0.05), miR-612 transfected (****P<0.0001) and combination (****P<0.0001) groups.

The regulation of PANC-1 cells with miR-612 mimic and 5-FU treatment also altered an immune checkpoint coding gene PD-L1. As shown in Figure 5(Fig. 5), PD-L1 relative expression was significantly decreased in all of the experimental groups. The changes were most significant in the combination group as it was in the former gene alterations.

### miR-612 significantly inhibited cell growth in combination with 5-FU

**Figure 6:**
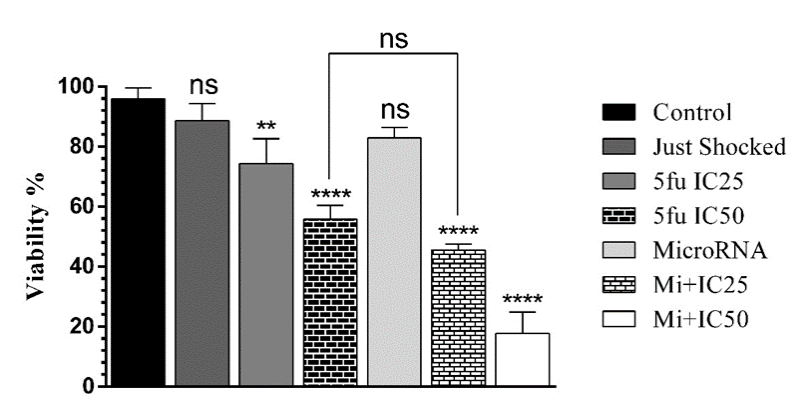
Transfection of miR-612 as a single agent does not affect proliferation significantly but as shown in both combination groups miR-612 and 5-FU reduces proliferation of the cells (****P<0.0001)

To perceive the viability of PANC-1 cells after administration of miR-612 mimic and 5-FU, MTT assay was executed. As illustrated in Figure 6(Fig. 6), no significant proliferation change was observed after solo miR-612 transfection. Then again, the combination group of the experiment showed a significant reduction in PANC-1 cell viability.

### miR-612 in combination with 5-FU induced apoptosis in PANC-1 cells

**Figure 7:**
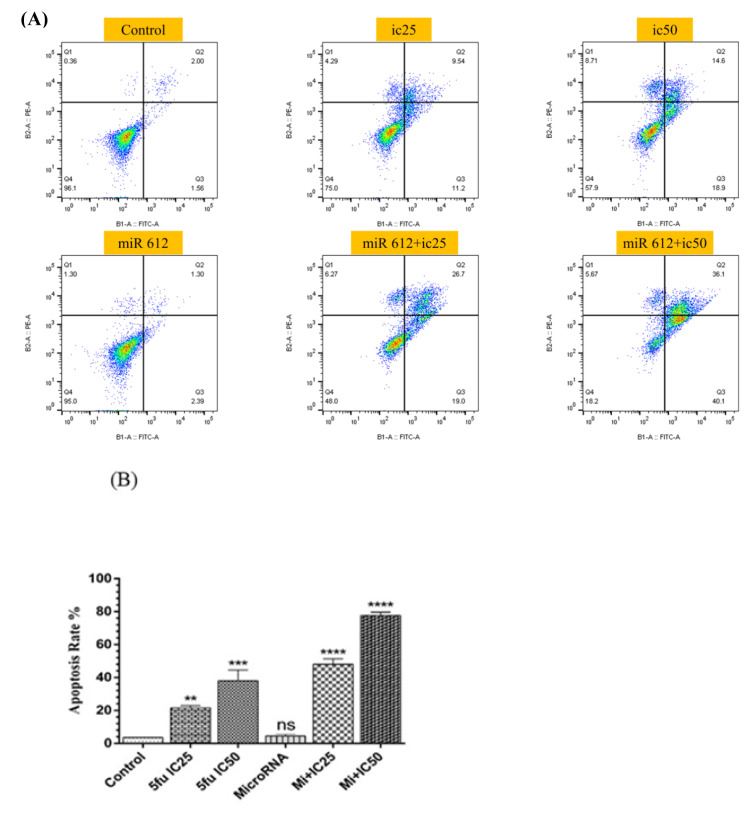
(A) Flow cytometry chart of the results assessed by FlowJo software. (B) miR-612 and 5-FU administration on PANC-1 cells did lead to an uprising in the apoptosis rate (****P<0.0001).

To confirm our cell viability assay, the results attained from flow cytometry demonstrated that miR-612 mimic transfection alone did not significantly increase the apoptosis rate yet its combination with both concentrations of 5-FU increased the rate of apoptosis in the PANC-1 cell line (Figure 7(Fig. 7)).

### miR-612 and 5-FU led to nuclear fragmentation in 5-FU cells

**Figure 8:**
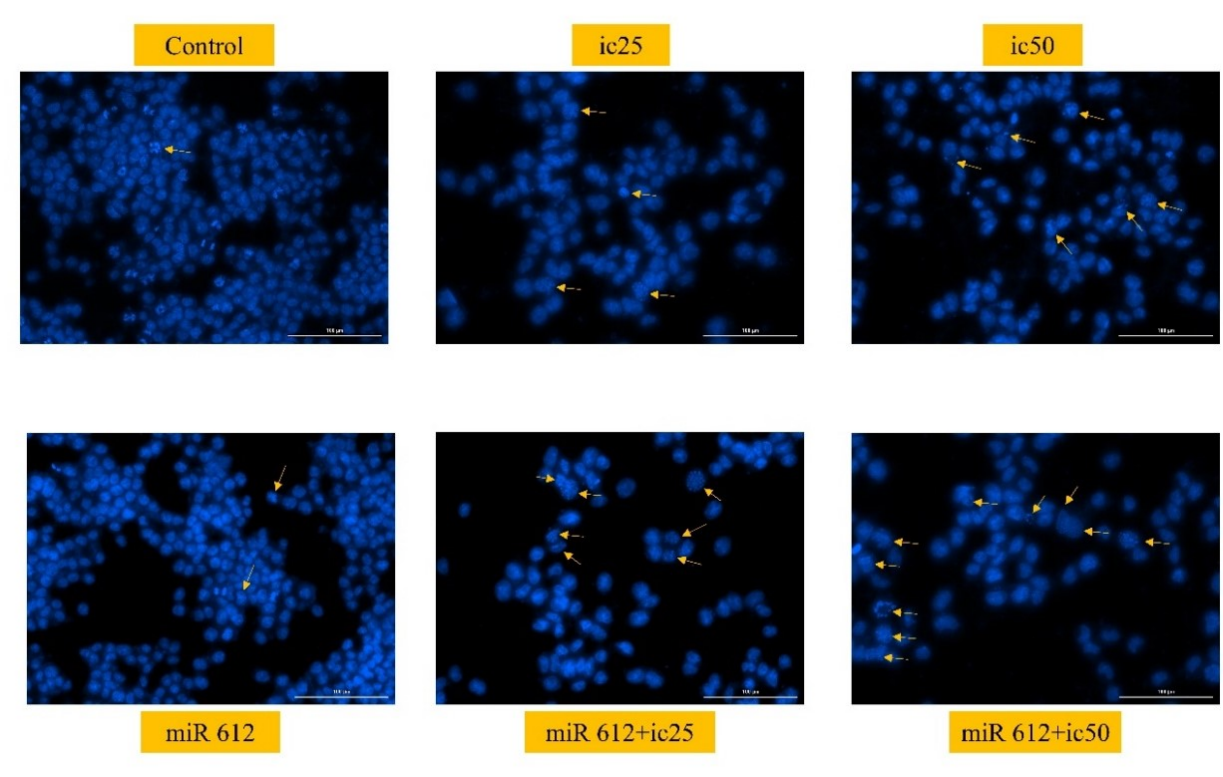
Captured moments of nuclear fragmentation of PANC-1 cells after DAPI staining. The fragmentation is mostly observed in the miR-612 and IC_50_ of the 5-FU group.

As a qualitative apoptosis test, the results of DAPI staining also showed promising results in the form of nuclear fragmentation. As illustrated in Figure 8(Fig. 8), the fragmented nuclei are mostly seen in the combination groups, rather than the miR-612 group.

### miR-612 inhibited migration of PANC-1 cells both in combination with 5-FU and alone

**Figure 9:**
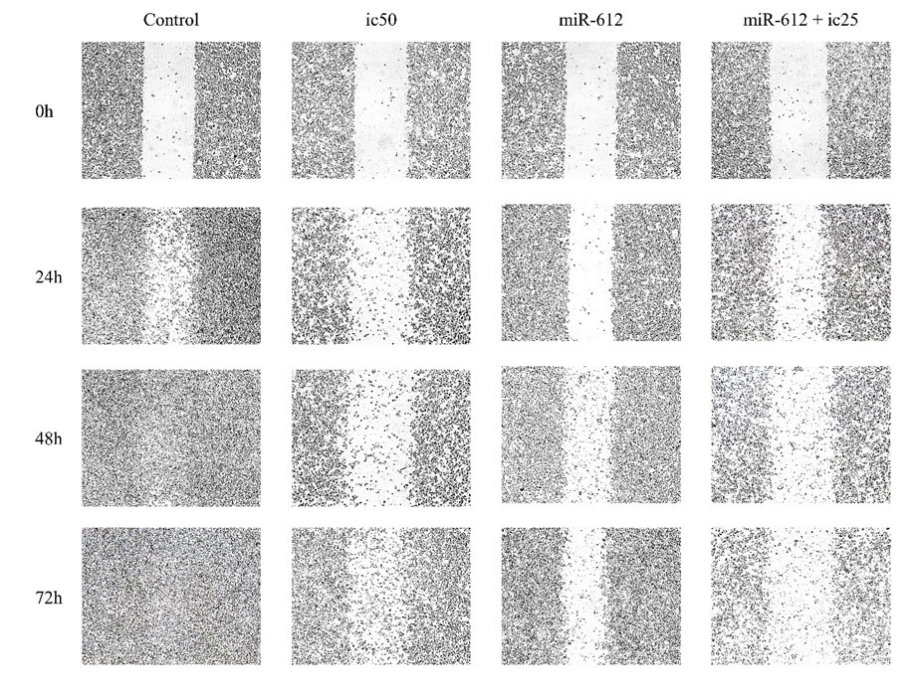
The inhibitory effect of miR-612 and 5-FU on PANC-1 cells' *in vitro* migration. A scratch was made by a yellow pipette tip and the gap between cells was captured by an invert microscope.

To perceive PANC-1 cell migration, a scratch test (wound healing assay) was conducted. As shown in Figure 9(Fig. 9), compared to the control group, mimic transfection of miR-612 in PANC-1 cells, caused an obvious inhibition of cell migration in both miR-612 and combination groups. The inhibition effect of miR-612 is even more compared to the IC_50_ of 5-FU.

### miR-612 mimic in combination with 5-FU leads to cell cycle arrest in the sub G1 phase

**Figure 10:**
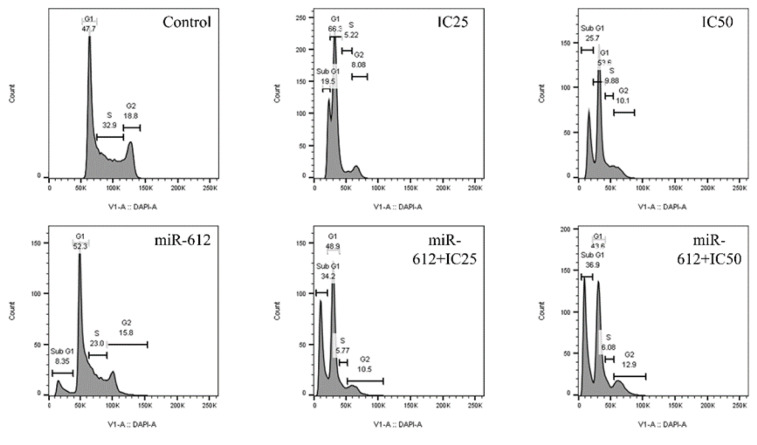
Sub G1 arrest is perceived in all groups compared to the control group. The least (8.35%) belongs to the miR-612 transfected group and the most (36.9%) belongs to miR-612 in combination with IC_50_ of 5-FU.

To obtain the effect of miR-612 mimic and 5-FU both as single agents and in combination with one another, we performed another flow cytometry test to attain cell cycle arrest in PANC-1 cells. As shown in Figure 10(Fig. 10), sub G1 arrest is observed in all groups except for the control group and the highest rate of sub G1 arrest has been assessed by miR-612 and IC_50_ of 5-FU.

### miR-612 inhibited colony formation of PANC-1 cells 

**Figure 11:**
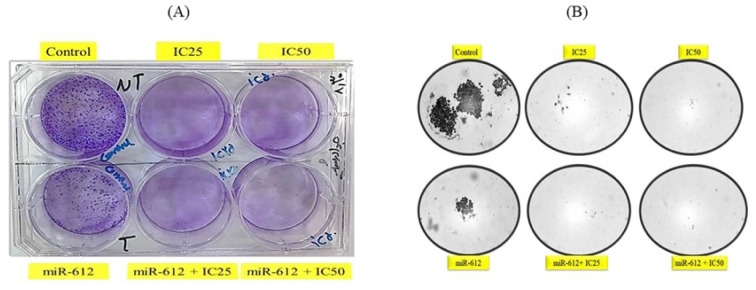
(A) A picture of colony formation plate. (B) Microscopic pictures of colony formation. Nearly no colonies are observed in 5-FU and combination groups.

The results of our colony formation showed more interesting results of the effect of miR-612 on the growth of the cells not only in combination with 5-FU but acting as a single mediator. As demonstrated in Figure 11(Fig. 11), miR-612 alone inhibited colony formation severely. Also, the drug control group and combination groups show nearly zero colonies.

See also Supplementary Data.

## Discussion

PANC-1 and Mia PaCa-2 cell lines that were used in this study have been exploited by other scientists in order to investigate pancreatic cancer as well. In the present experiment, as a first step, the expression of miR-612 in the mentioned cell lines was measured using qRT-PCR following specified cDNA synthesis. Downregulation of miR-612 in the PANC-1 cells after the transfection of its mimic was a promising result due to the downregulation of this very same microRNA in other cancers including HCC, CRC, ovarian and cervical cancers, and melanoma (Sheng et al., 2015[20]; Yu et al., 2018[27]; Jin et al., 2020[10]; Tang et al., 2014[24]; Zhu et al., 2018[28]). The findings of this study and previous researches suggest that miR-612 could be a tumor suppressor microRNA in various cancers. Likewise, a significant growth and migration inhibition was observed after the transfection of miR-612 on PANC-1 cells which promoted the hypothesis of this study.

To evaluate the cytotoxic effects of miR-612 and 5-FU in combination, MTT assay was utilized and the results showed a great deal of reduction inefficient dosage of the chemotherapeutic agent. The viability of the cells was decreased to less than 20 % as a result of combining miR-612 and 5-FU. Another study carried out in 2014 investigated the role of miR-612 in combination with cisplatin (another frequently used chemotherapeutic agent) on hepatocellular carcinoma cells and the outcome was a decrease in stemness and tumor size and drug resistance (Tang et al., 2014[24]). The result of the latter study was rather confirming the outcomes of the present experiment.

BAX, BCL-2, Caspase-3, MMP9, and PD-L1 were target genes of this study according to their history in related literature.

BAX gene encodes a protein with the same name which is pro-apoptotic and has an important role in the apoptosis process. According to the results of the qRT-PCR, after combinatorial usage of miR-612 and 5-FU on PANC-1 cells significant overexpression of BAX gene was observed. Earlier in the year 2004 von Haefen and his team measured the BAX gene expression and its synergism with cellular death ligand TRAIL and 5-FU. Results were a sign of the fact that the synergism between 5-FU and TRAIL completely depends on BAX expression in prostate cancer cell lines. In the presence of BAX gene, 5-FU, and TRAIL successfully induced apoptosis on the cell lines (von Haefen et al., 2004[25]). According to our study and the discussed experiment miR-612 in combination with 5-FU could target the pro-apoptotic pathway in pancreatic cancer and the results of the apoptosis test also confirm the notion showing more than 70 % apoptosis in the combination group of the cells.

On the contrary, BCL-2 is an anti-apoptotic gene and has shown a significant expression decrease in the combination group of this experiment. As a confirmation, a study conducted earlier this year demonstrated that overexpression of miR-612 in human cardiomyocyte cell line decreased the protein and mRNA levels of BCL-2 and increased the protein and mRNA levels of BAX which is very similar to our results considering gene expression levels (Li et al., 2020[13]).

MMP9 gene is a member of the matrix metalloproteinase family and involves in metastasis of cancer cells. The outcomes of qRT-PCR showed a significant decrease in the expression of MMP9 gene following transfection and treatment with miR-612 and 5-FU in PANC-1 cells. Previous research showed that transfection of miR-612 to the T24 bladder cancer cell line, halters invasion, and EMT and Western blot analysis showed that EMT related markers such as MMP9 were downregulated in the miR-612 transfected group (Liu et al., 2018[14]). Furthermore, our wound healing assay results also confirmed the fact that a combination of miR-612 and 5-FU inhibits migration and possibly metastasis as well. The gap between the combination group cells was wider than other groups according to the microscopic pictures of our scratch test.

Another apoptotic target gene of the experiment was caspase 3 which has an important role in the apoptotic cascade alongside caspases 8 and 9. A significant increase in Cas-3 expression was observed in the combination group which is another confirmation of the fact that miR-612 activates the apoptotic pathway in the PANC-1 cells. In one of the pre-mentioned studies, after transfection of miR-612 to the human cardiomyocyte cell line, there were increased mRNA and protein levels of Cleaved Cas-3 which was another indicator of apoptosis induction by miR-612 (Li et al., 2020[13]). In another study, after miR-612 transfection to the clinical samples and cell lines of NF1, a significant increase in caspases 3, 8 and 9 protein levels was observed (Wang et al., 2019[26]).

The last gene investigated as a target gene for miR-612 was PD-L1 which encodes an immune checkpoint protein with the same name and is usually overexpressed in tumor cells. Transfection of miR-612 both singly and in combination with 5-FU treatment decreases the expression of PD-L1 in comparison to the control group. In a previous study, PD-L1 and vimentin expression increased in the pancreatic cancer cell lines compared to the control samples (Song et al., 2014[22]) and another study also showed that in lung cancer clinical samples, overexpression of PD-L1 leads to overexpression of vimentin as well (Shimoji et al., 2016[21]). According to the fact that both MMP9 and vimentin are metastatic genes, their downregulation is related to inhibition of metastasis and migration and since the link between PD-L1 and vimentin has been shown in previous researches, we could assume that a decrease in PD-L1 expression could also mean metastasis inhibition in PANC-1 cells. This could be the beginning of using PD-L1 checkpoint inhibitor alongside miR-612 mimic and 5-FU as a combinatorial therapy modality in the treatment of pancreatic cancer.

As discussed before, the results of our wound healing assay showed the inhibitory effect of miR-612 on the migration of PANC-1 cells. Another study also showed that miR-612 inhibits cell migration and invasion in lung cancer cell lines (Kang et al., 2019[11]).

A former study used the colony formation test to evaluate the suppression effect of miR-612 on the bladder cancer cell line. After the transfection of miR-612 on the T4 cells, a noticeable reduction was assessed in the number of colonies (Liu et al., 2018[14]). Our results confirmed this suppression effect of miR-612 on the PANC-1 colony formation. A reduction near to zero colonies was observed in the miR-612 transfected cells both alone and in combination with 5-FU.

## Conclusion

Pancreatic cancer is one of the most prevalent cancers related to the digestive system and as mentioned before, 90 % of the cases are PDAC which originates from the exocrine part of the pancreas and its progress leads to metastasis to the liver and other organs. 5-fluorouracil (5-FU) is one of the most routine chemotherapeutic agents used for pancreatic cancer treatment and has been used in other studies in combination with other drugs and even recombinant therapies. MicroRNA replacement therapy is one of the novel modalities in the treatment of various cancers and in case of proper function, has more sufficiency and fewer side effects compared to chemotherapy. The tumor suppressor role of miR-612 was previously reported in other cancers and according to our qRT-PCR results, this particular miRNA is also downregulated in pancreatic cancer cells *in vitro*. To evaluate the simultaneous effect of miR-612 in combination with 5-FU we cultured PANC-1 cells in different groups and according to the results of apoptosis, cell cycle, colony formation, DAPI and wound healing assays the inhibitory effect of miR-612 and 5-FU reached their greatest amount in combination groups. Additionally, the relative expression level of apoptosis-related genes such as BAX and Caspase 3 in combination groups were in their summits. Finally, analyzing these results we can assume that miR-612 could be a therapeutic target in the treatment of pancreatic cancer especially in combination therapies. Yet, to approve the hypothesis discussed in this study additional data and more *in vitro *and* in vivo* experiments are needed. 

## Notes

Reza Mohammadzadeh and Behzad Baradaran (Immunology Research Center, Tabriz University of Medical Sciences, Iran; Tel: +98 413 337 1440, E-mail: behzad_im@yahoo.com) contributed equally as corresponding authors.

## Supplementary Material

Supplementar data

## Figures and Tables

**Table 1:**
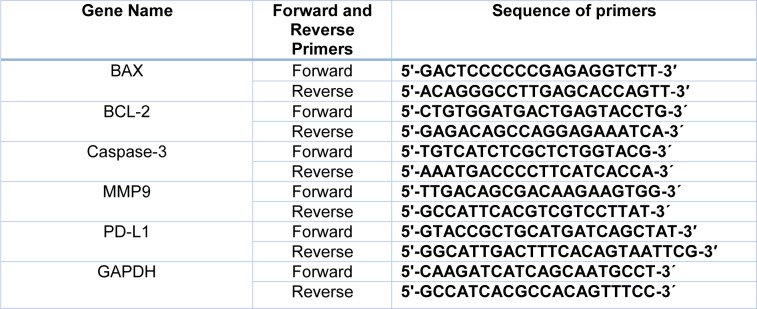
Sequences of primers
