# Supplementary material for: Simultaneous microRNA-612 restoration and 5-FU treatment inhibit the growth and migration of human PANC-1 pancreatic cancer cells
Source: EXCLI J. 2021 Jan 21;20:160–73. doi: 10.17179/excli2020-2900 (PMC7868639; doi:10.17179/excli2020-2900)
Supplement: Supplementar data [file EXCLI-20-160-s-001.pdf]

EXCLI Journal 2021;20:160-173 – ISSN 1611-2156
Received: September 12, 2020, accepted: January 18, 2021, published: January 21, 2021

Supplementary data to:

Original article:

SIMULTANEOUS MICRORNA-612 RESTORATION AND 5-FU TREATMENT INHIBIT THE GROWTH AND MIGRATION OF HUMAN PANC-1 PANCREATIC CANCER CELLS

Darya Javadrashid[1,2], Reza Mohammadzadeh[2*], Amir Baghbanzadeh[1], Sahar Safaee[1], Mohammad Amini[1], Ziba Lotfi[1], Elham Baghbani[1], Vahid Khaze Shahgoli[1], Behzad Baradaran[1*]

[1] Immunology Research Center, Tabriz University of Medical Sciences, Iran
[2] Faculty of Basic Sciences, Department of Biology, University of Maragheh, Iran

* **Corresponding authors:** Behzad Baradaran, Immunology Research Center, Tabriz University of Medical Sciences, Iran. Tel: +98 413 337 1440; E-mail: behzad_im@yahoo.com
Reza Mohammadzadeh, Faculty of Basic Sciences, Department of Biology, University of Maragheh, Iran. Tel: +989381983364; E-mail: rmohamadzadeh82@gmail.com

https://orcid.org/0000-0002-8642-6795 (Behzad Baradaran)
https://orcid.org/0000-0002-7893-5151 (Reza Mohammadzadeh)

http://dx.doi.org/10.17179/excli2020-2900

This is an Open Access article distributed under the terms of the Creative Commons Attribution License (http://creativecommons.org/licenses/by/4.0/).

Supplementary Table 1: Raw data showing investigated genes' expression after transfection of PANC-1 cells with the miR-612 or their corresponding control group. The relative expression of each gene was analyzed by comparative threshold cycle (Ct). Ct value was normalized using the formula ΔCt = Ct (investigated genes) - Ct (SNORD and GAPDH). Then formula ΔΔCt = ΔCt (treated) - ΔCt (control) was used. Finally, the formula 2−ΔΔCt was used for estimating relative expression of each gene.

Group	CT values SNORD				CT values miR-612				Fold induction				
**PANC1**	R1	R2	R3	mean	R1	R2	R3	mean	R1	R2	R3	mean	SD
**Dose 60**	25.87	25.75	26.2	25.94	29.83	29.42	29.42	29.42	156.498	164.2785	147.0334	155.9366	7.051464
**Dose 80**	25.53	25.52	25.81	25.62	28.09	27.81	27.81	27.81	413.0006	427.565	415.8732	418.8129	6.298781
**Dose 100**	26.29	26.28	25.83	26.13333	28.91	28.7	28.7	28.7	396.1766	390.7224	385.3432	390.7474	4.422752
**Control**	25.7	25.89	25.71	25.76667	36.95	36.92	36.92	36.92	1	1	1	1	0
	CT values SNORD				CT values miR-612				Fold induction				
**PANC1**	R1	R2	R3	mean	R1	R2	R3	mean	R1	R2	R3	mean	SD
**24h**	23.31	23.78	23.01	23.36667	26.04	26.26	26.03	26.11	367.0925	380.038	352.1387	366.4231	11.39969
**48h**	26.22	26.38	26.43	26.34333	28.74	28.68	29.16	28.86	424.6116	430.539	430.539	428.5632	2.794183
**72h**	27.18	27.31	27.04	27.17667	31.44	31.43	31.45	31.44	127.1158	121.9377	134.3637	127.8057	5.096325
**Control**	25.7	25.87	25.71	25.76	36.95	36.92	37.19	37.02	1	1	1	1	0
	CT values GAPDH				CT values BAX				Fold induction				
**PANC1**	R1	R2	R3	mean	R1	R2	R3	mean	R1	R2	R3	mean	SD
**5FU**	19.95	19.82	20.13	19.96667	26.3	26.1	26.32	26.24	2.42839	2.297397	2.114036	2.279941	0.128927
**miR**	20.51	20.89	21.07	20.82333	27.82	28.2	27.98	28	1.248331	1.125058	1.283426	1.218938	0.067912
**miR+5FU**	20.23	20.46	20.88	20.52333	26.24	26.24	26.42	26.3	3.07375	3.24901	3.317278	3.213346	0.102568
**Control**	21.67	22.03	21.88	21.86	29.3	29.51	29.15	29.32	1	1	1	1	0
	CT values GAPDH				CT values CAS3				Fold induction				
**PANC1**	R1	R2	R3	mean	R1	R2	R3	mean	R1	R2	R3	mean	SD
**5FU**	19.95	19.82	20.13	19.96667	32.27	31.45	31.62	31.78	30.06473	35.0174	33.59093	32.89102	2.081609
**miR**	20.51	20.89	21.07	20.82333	35.91	35.48	35.7	35.69667	3.555371	4.500234	3.810552	3.955386	0.399102
**miR+5FU**	20.23	20.46	20.88	20.52333	30.9	30.54	30.82	30.75333	94.35323	102.5369	98.36001	98.41673	3.341228
**Control**	21.67	22.03	21.88	21.86	38.9	38.79	38.44	38.71	1	1	1	1	0

EXCLI Journal 2021;20:160-173 – ISSN 1611-2156
Received: September 12, 2020, accepted: January 18, 2021, published: January 21, 2021

Supplementary Table 1 (cont.): Raw data showing investigated genes' expression after transfection of PANC-1 cells with the miR-612 or their corresponding control group. The relative expression of each gene was analyzed by comparative threshold cycle (Ct). Ct value was normalized using the formula ΔCt = Ct (investigated genes) - Ct (SNORD and GAPDH). Then formula ΔΔCt = ΔCt (treated) - ΔCt (control) was used. Finally, the formula 2−ΔΔCt was used for estimating relative expression of each gene.

**Group**	CT values GAPDH				CT values BCL2				Fold induction				
**PANC1**	R1	R2	R3	mean	R1	R2	R3	mean	R1	R2	R3	mean	SD
**5FU**	19.95	19.82	20.13	19.96667	34.45	32.31	32.96	33.24	0.051119	0.086569	0.115824	0.084504	0.026456
**miR**	20.51	20.89	21.07	20.82333	30.9	30.32	31.14	30.78667	0.882703	0.721965	0.784584	0.796417	0.066152
**miR+5FU**	20.23	20.46	20.88	20.52333	36.22	38.54	40.3	38.35333	0.018199	0.001797	0.001202	0.007066	0.007876
**Control**	21.67	22.03	21.88	21.86	31.88	30.99	31.6	31.49	1	1	1	1	0
	CT values GAPDH				CT values MMP9				Fold induction				
**PANC1**	R1	R2	R3	mean	R1	R2	R3	mean	R1	R2	R3	mean	SD
**5FU**	19.95	19.82	20.13	19.96667	39.36	39.46	39.05	39.29	0.285191	0.351111	0.438303	0.358202	0.062708
**miR**	20.51	20.89	21.07	20.82333	39.21	40.33	39.68	39.74	0.466516	0.403321	0.543367	0.471068	0.057264
**miR+5FU**	20.23	20.46	20.88	20.52333	41.77	42.03	43.34	42.38	0.065154	0.092142	0.037681	0.064992	0.022234
**Control**	21.67	22.03	21.88	21.86	39.27	40.16	39.61	39.68	1	1	1	1	0
	CT values GAPDH				CT values PD-L1				Fold induction				
**PANC1**	R1	R2	R3	mean	R1	R2	R3	mean	R1	R2	R3	mean	SD
**5FU**	19.95	19.82	20.13	19.96667	33.34	33.12	33.77	33.41	0.895025	0.742262	0.707107	0.781465	0.081572
**miR**	20.51	20.89	21.07	20.82333	34.73	34.54	35.34	34.87	0.503478	0.582367	0.456916	0.514253	0.051779
**miR+5FU**	20.23	20.46	20.88	20.52333	35.75	34.84	35.84	35.47667	0.204476	0.351111	0.283221	0.279603	0.059918
**Control**	21.67	22.03	21.88	21.86	34.9	34.9	35.02	34.94	1	1	1	1	0
	CT values SNORD				CT values miR612				Ratio				
	R1	R2	R3	mean	R1	R2	R3	mean	R1	R2	R3	mean	SD
**PANC1**	26.4	26.89	26.21	26.5	33.36	33.71	32.91	33.3267	0.00803	0.008851	0.009618	0.00881	0.000649
**Mia paca2**	24.56	24.81	25.02	24.79667	29.06	29.52	29.98	29.52	0.044	0.038	0.032	0.038	0.004899

EXCLI Journal 2021;20:160-173 – ISSN 1611-2156
Received: September 12, 2020, accepted: January 18, 2021, published: January 21, 2021

Supplementary Table 2: Raw data of MTT assay analysis showing the capacity of cell proliferation of the miR-612-transfected cells and 5-FU treated groups in comparison with the control group

8000 cell/well	OD1	OD2	OD3
control	100.000	92.730	94.880
Just shocked	88.005	83.350	94.850
5-FU IC25	83.76402	70.7947	68.2225
5-FU IC50	60.2231	51.0365	56.0702
miR-612	81.1896	86.8844	80.5948
miR-612+IC25	43.83715	47.78157	44.96831
miR-612+IC50	15.92394	11.53584	25.56330
